# Genetic characterization of 11 porcine reproductive and respiratory syndrome virus isolates in South China from 2014 to 2015

**DOI:** 10.1186/s12985-017-0807-4

**Published:** 2017-07-24

**Authors:** Linyang Yu, Pandeng Zhao, Jianguo Dong, Yanling Liu, Leyi Zhang, Pengshuai Liang, Lei Wang, Changxu Song

**Affiliations:** 1 College of Animal Science & National Engineering Center for Swine Breeding Industry, South China Agriculture University, Guangzhou, 510642 China; 2 Henan University of Animal Husbandry and Economy, Zhengzhou, 450046 China; 3 College of Animal Husbandry and Veterinary, Xinyang Agriculture and Forestry University, Xinyang, 464000 China

**Keywords:** PRRSV, Phylogenetic analysis, NSP2, GP5, Mutation

## Abstract

**Background:**

Porcine reproductive and respiratory syndrome (PRRS) has leaded to an enormous loss per year to the swine industry, its etiology porcine reproductive and respiratory syndrome virus (PRRSV) is a highly mutated virus in pigs. To fully understand the genetic characteristics of PRRSV genome in South China, this study collected the lung samples infected with PRRSV in Guangdong and Hainan province from 2014 to 2015 and tried to isolate the PRRSV. Finally, the complete genomes of isolated strains were sequenced and analyzed.

**Methods:**

Virus isolation was performed in MARC-145 cells. The 13 fragments of PRRSV genome were amplified by RT-PCR and the complete PRRSV genome sequence was obtained by SeqMan program of DNASTAR7.0 software. Nucleotide and deduced amino acid (AA) sequences of NSP2 and ORF5 were aligned using the MegAlign program of DNASTAR7.0 software to determine sequence homology. A phylogenetic tree was constructed using MEGA5.2 software with the neighbor-joining method to analyze the evolutionary relationship.

**Results:**

11 PRRSV strains were isolated in South China from 2014 to 2015. All the isolated strains clustered into subgenotype V along with the HP-PRRSV representative strains JXA1, HuN4 and JXwn06. The subgenotype V was furtherly divided into two groups. AA sequence alignment analysis indicated that all the isolated strains had 1 AA deletion and 29 AA continuous deletion at position 481 and 533-561. Notably, GDHY strain had another 120 AA continuous deletion at position 629-748. All the isolated strains had an A137S mutation in the residue A137 of GP5 which was considered to differentiate vaccine strains. All the isolated strains had a L39I mutation in the primary neutralizing epitope (PNE) of GP5. Except GDHZ had a N34T mutation, all the other isolated strains had conserved N30, N44 and N51 glycosylation sites in the four potential N-glycosylation sites (N30, N34, N44 and N51) of GP5.

**Conclusions:**

Our study showed that the prevalent strains in this region were highly pathogenic PRRS virus-like. Moreover, one new strain having another 120 amino acids continuous deletion except the discontinuous 30 (29+1) amino acids deletion in NSP2 region had emerged. Besides, the isolated strains had extensive amino acids substitutions in the putative signal, extravirion and intravirion regions of GP5. These results showed that PRRSV has undergone extensive variation in South China, providing some theoretical basis for researching effective vaccince to better controling the PRRSV in this area.

## Background

Porcine reproductive and respiratory syndrome (PRRS) is an important swine contagious disease across the world, leading to an enormous loss per year to the swine industry [1]. In 1987, PRRSV was first reported in the United States, and later it appeared in Europe [2, 3]. Unfortunately, after a short while, PRRS also outbroke in Asia countries. In 2006, a highly pathogenic strain of porcine reproductive and respiratory syndrome virus (HP-PRRSV) broke out in China and spreaded rapidly to most areas of China and neighboring countries [4–6]. Recently, mang NADC30 like strains had been monitored and isolated in the Middle, North-east and South-east China [7–9].

Porcine reproductive and respiratory syndrome virus (PRRSV) is an enveloped, positive-sense single-stranded RNA virus belonging to the family Arteriviridae, order Nidovirales [10]. The PRRSV complete genome is about 15 kb in length, including at least 10 open reading frames (ORFs): ORF1a, ORF1b, ORF2a, ORF2b, ORF3, ORF4, ORF5, ORF5a, ORF6 and ORF7 [11, 12]. ORF1a and ORF1b encode viral replicase polyproteins, which are furtherly cleaved into 16 nonstructural proteins (Nsps), including NSP1α, NSP1β, NSP2, NSP2TF, NSP2N, NSP3, NSP4, NSP5, NSP6, NSP7α, NSP7β, NSP8, NSP9, NSP10, NSP11 and NSP12 [13–15], whereas other ORFs encode the viral structural proteins GP2a, E, GP3, GP4, GP5, ORF5a, M, and N, respectively (12).

To fully understand the genetic characteristics of PRRSV genome in South China, we collected the lung samples infected with PRRSV in Guangdong and Hainan province from 2014 to 2015 and tried to isolate the PRRSV. Finally, 11 PRRSV strains were successfully isolated and the complete genomes were sequenced and analyzed.

## Methods

### Clinical samples

Lung samples were collected from sick pigs infected with PRRSV in Guangdong and Hainan provinces of South China from 2014 to 2015. The lung samples were homogenized and centrifuged and the supernatants were used for virus isolation. All the samples were collected according to the animal ethical regulation of National Engineering Center for Swine Breeding Industry (NECSBI 2015–16).

### Virus isolation

Virus isolation was performed in MARC-145 cells which were maintained in Dulbecco’s Modified Eagle Medium (DMEM) containing 10% fetal bovine serum (FBS; Thermo), 100 mg/mL penicillin, and 100 units/mL of streptomycin. MARC-145 cells seeded in 6-well cell culture plates (Corning Inc., USA) were incubated with the supernatants from the homogenized lung samples for 1 h, then the supernatants were discarded and DMEM was added into the 6-well cells and the cells were maintained at 37 °C with 5% CO_2_. The cultured cells and supernatants were harvested when cytopathic effect (CPE) appeared in 70% and the recovered strains were passaged twice in MARC-145 cells and the viral cultures of the third passage were used for genomic sequence analysis.

### Primers design

To determine the full genome sequence of isolated strains, primers were designed based on the referenced PRRSV sequences available in NCBI. The primers used for complete genome sequencing were given in Table 1.

**Table 1:** Primers used for PRRSV genome amplification. The primers for amplifying PRRSV genome were listed in Table 1

Primers Name	Sequence(5′-3′)	Position^a^
PRRSV1F	ATGACGTATAGGTGTTGGCT	1–20
PRRSV1R	GTCGCACCAGAGCGTGCTTTC	1357–1377
PRRSV1F	CAGAATCAGGGTTGAGCCCAAT	1255–1276
PRRSV1R	CTGCCCAGGCCATCATTTCTGAA	2551–2573
PRRSV2F	CCACTGGACTTGGCCCGCGAC	2451–2471
PRRSV2R	CTGAAGGCAGCAAATCAGTGA	3780–3800
PRRSV3F	CCGGTCTGCGACCAACCTGCC	3680–2700
PRRSV3R	GAAAATACACCCAAGAGGGGAG	4029–4050
PRRSV4F	CACACCTCTTCAAATCTGACAG	3921–3942
PRRSV4R	CGAAGGCATATTTACAGAAATC	5368–5389
PRRSV5F	CCCTTACCTGGTTGCTTTGTGT	5280–5301
PRRSV5R	GTAACGGATGCCCTTGAGTTGC	6521–6542
PRRSV6F	GGATGTTTGTGCTATCTTGGCT	6378–6399
PRRSV6R	GCGGCTAGCAGTTTAAACACTGC	7684–7706
PRRSV7F	GAGCAAGCCCTTGGTATGATGA	7583–7604
PRRSV7R	CAAGGCACCTGCCTAAAACCGGA	8844–8866
PRRSV8F	CGTTGAGTGGTGTCACCCAGGG	8756–8777
PRRSV8R	TTCCCTCCTGGATGAAGCAGCG	10,281–10,302
PRRSV9F	GGCTTTGGGGACGTGCCGGTTC	10,164–10,185
PRRSV9R	AACTGATTCCTTGGGAAGGAAG	11,400–11,421
PRRSV110F	CTGAGTCCCTCCCACATGCCTT	11,312–11,333
PRRSV110R	AAGTACTATTATACACTATG	12,613–12,632
PRRSV11F	TGGATGTGGTGGCTCATTTTC	12,498–12,518
PRRSV11R	CCCCAACATACTTGAACATTCA	13,776–13,797
PRRSV12F	CCAACATGTCAAGGAGTTTAC	13,646–13,666
PRRSV12R	CTTTCGCTGCTTGCCGTTGTTA	14,898–14,919
PRRSV13F	TACGGTTAACGGCACATTGGTG	14,798–14,819
PRRSV13R	d(T)20AATTTCGGCCGCATGG	15,395–15,432

^a^Numbers represents the nucleotide position within the genome of CH-1a (GenBank accession number: AY032626)

### RNA extraction and RT-PCR

Total RNA was extracted using TRIzol reagent (Life Technologies, USA) according to the manufacturer’s instructions. Reverse transcription was performed in a total volume of 20 μL containing 10.5 μL total RNA, 4 μL 5× reverse transcription buffer, 2 μL deoxynucleoside triphosphate (dNTP) mixture (10 mM), 1 μL 9-mer random primers (50 pM), 2 μL reverse transcriptase M-MLV (Takara, Dalian), and 0.5 μL RNase inhibitor (40 U/μL). The reactants were mixed gently, placed in a water bath at 42 °C for 1 h, then incubated on ice for 2 min. The polymerase chain reaction was conducted using PrimeSTAR HS DNA Polymerase (Takara, Dalian).

### Nucleotide cloning and sequencing

PCR products were purified using the Wizard SV Gel and PCR Clean-Up system (Promega, USA), and then cloned into pEASY Simple Blunt vector (TransGen tech Co., Beijing, China). Plasmids were submitted to BGI (Guangzhou, China) for sequencing and the complete PRRSV genome sequence was obtained by SeqMan program of DNASTAR7.0 software (DNASTAR Inc., Madison, WI, USA). The complete genome sequence was submitted to GenBank and the accession no. was listed in Table 2.

**Table 2:** Information of 11 PRRSV isolated from South China. The designation, isolated year, accession No, sample, area of isolated strains were listed in Table 2

No.	Designation	Isolated year	Accession No.	Sample	Area
1	GDGZ	2014	KY488471	Lung	Guangzhou
2	GDJM	2014	KY488470	Lung	Jiangmen
3	GDMM	2014	KY488472	Lung	Maoming
4	GDST	2014	KY498542	Lung	Shantou
5	GDZQ	2014	KY488473	Lung	Zhaoqing
6	HNHK1	2014	KY488474	Lung	Haikou
7	HNHK2	2014	KY488475	Lung	Haikou
8	GDHY	2015	KY488476	Lung	Heyuan
9	GDHZ	2015	KY488477	Lung	Huizhou
10	GDQY	2015	KY488478	Lung	Qingyuan
11	GDSG	2015	KY488479	Lung	Shaoguan

### Sequence alignment and phylogenetic analysis

Nucleotide and deduced amino acid (AA) sequences were aligned using the MegAlign program of DNASTAR7.0 software (DNASTAR Inc., WI, USA) to determine sequence homology. A phylogenetic tree was constructed using MEGA5.2 software with the neighbor-joining method; bootstrap values were calculated for 1000 replicates for alignment with multiple sequences of representative PRRSV sequences available in GenBank (Table 3).

**Table 3:** Information of the representative strains. The strain designation, area, isolatedtime, accession No. of representative strains were listed in Table 3

No.	Strain	Area	Time	Accession No
1	EDRD-1	Japan	1992	AB288356
2	Leystad virus	Netherlands	1993	M96262
3	CH-1a	Beijing, China	1996	AY032626
4	BJ-4	Beijing, China	1996	AF331831
5	PL97–1	South Korea	1997	AY585241
6	HB-1(sh)/2002	Hebei, China	2002	AY150312
7	VR-2332	America	2003	AY150564
8	Resp PRRS MLV	America	2005	AF066183
9	SHB	Guangdong, China	2005	EU864232
10	JXA1	Jiangxi, China	2006	EF112445
11	JXwn06	Jiangxi, China	2006	EF641008
12	GD	Guangdong, China	2006	EU825724
13	HuN4	Hunan, China	2007	EF635006
14	CH-1R	Heilongjiang, China	2008	EU807840
15	JXA1-P80	Guangdong, China	2008	FJ548853
16	GDBY1	Guangdong, China	2008	GQ374442
17	NADC30	America	2008	JN654459
18	GD-100	Guangdong, China	2009	GU143913
19	QY2010	Guangdong, China	2010	JQ743666
20	GX1003	Guangxi, China	2010	JX912249
21	GM2	Guangdong, China	2011	JN662424
22	QYYZ	Guangdong, China	2011	JQ308798
23	SD16	Shanxi, China	2012	JX087437
24	JL580	Jilin, China	2013	KR706343
25	CHsx1401	Beijing, China	2015	KP861625

## Results

### Phylogenetic analysis of the isolated PRRSV genome

**Fig. 1:**
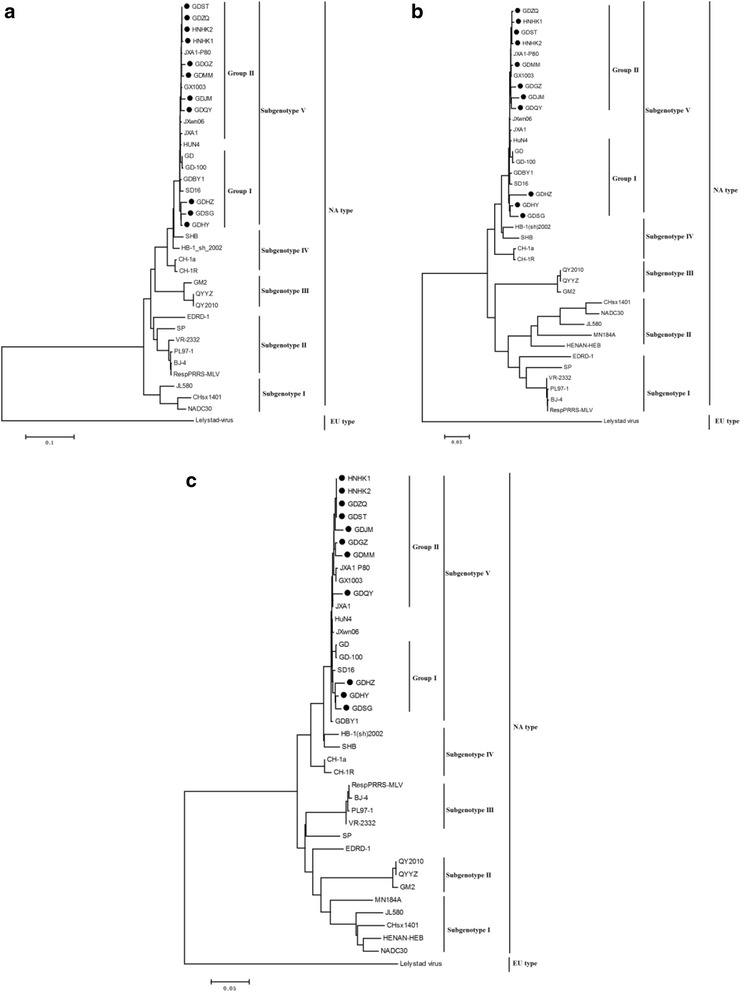
Phylogenetic trees based on the complete genome, NSP2 and ORF5 of PRRSV. **a** Complete genome; (**b**) NSP2 nucleotide; (**c**) Open reading frame5 (ORF5). The isolate identified in this study was indicated by *black dots*

To understand the evolution relationships of all the isolated PRRSV strains with the representative strains, phylogenetic trees were constructed using the neighor-joining method based on the complete genome, NSP2 nucleotide and ORF5 nucleotide sequences, respectively. As shown in Fig. 1a, the isolated strains and the representative strains could be divided into five subgenotypes: Subgenotype I, II, III, IV and V. All the isolated strains clustered into subgenotype V along with the HP-PRRSV representative strains JXA1, HuN4 and JXwn06. The subgenotype V was furtherly divided into two groups. GDHZ, GDHY and GDSG belonged to Group I, sharing a high homology with HP-PRRSV strains HuN4 and GD. The other isolated strains belonged to Group II, sharing a high identity with the HP-PRRSV strains JXA1 and JXwn06. When the phylogenetic trees were constructed based on NSP2 and ORF5 gene sequences of all the isolated strains and the reference strains, they had a similar cluster. GDHZ, GDHY and GDSG belonged to Group I, sharing a high homology with HP-PRRSV strains HuN4 and GD. The other isolated strains belonged to Group II, sharing a high identity with the HP-PRRSV strains JXA1 and JXwn06 (Fig. 1b and c).

### Alignment and analysis of NSP2 amino acid sequences

**Fig. 2:**
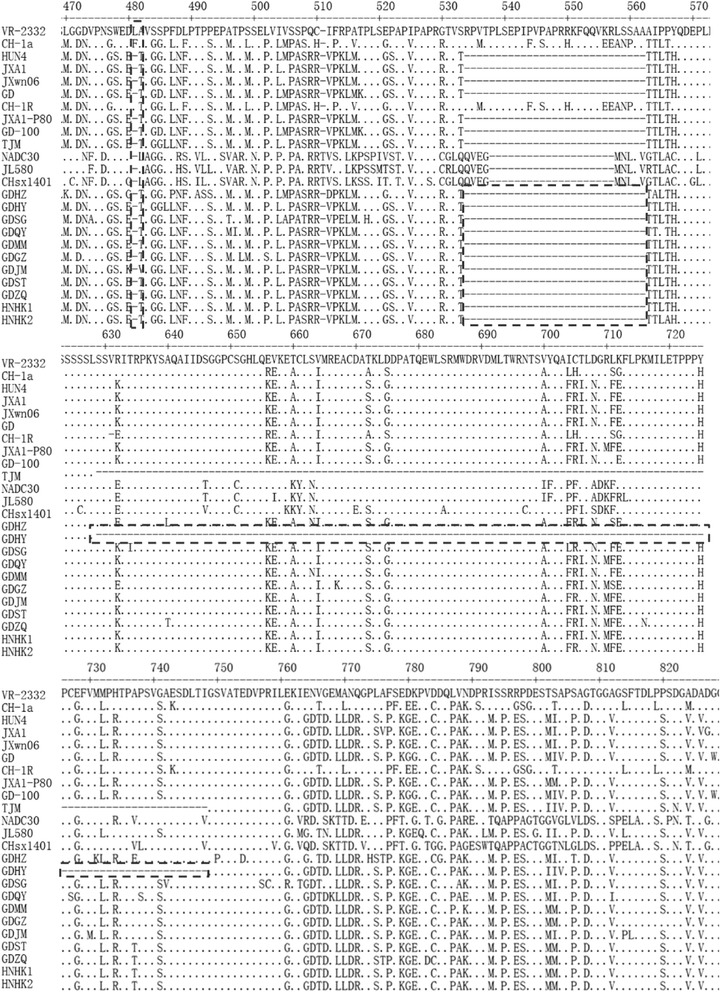
Alignment of the partial NSP2 animo acid sequences of isolated PRRSV strains. The deleted regions were indicated by a *dotted box*

To explore the genetic characteristics of the isolated PRRSV strains, the NSP2 AA sequence of isolated strains were analyzed. The results showed that AA sequence identities among all the isolated strains ranged from 92.3%–97.7%. All the NSP2 AA sequence of isolated strains shared 75.3%–78.0%, 84.8%–88.0%, and 93.7%–98.7% AA identity with the reference strains VR-2332, CH-1a and JXA1, respectively. Compared with the NADC30 and recently isolated NADC30-like strains JL580 and CHsx1401, they shared 66.4%–67.7% AA identity. However, they only shared 11.8%–13.2% AA identity with the European genotypic strain Lelystad virus (LV). These results indicated that all the isolated strains belonged to North American genotype. The AA sequence alignment analysis indicated that all the isolated strains had 1 AA deletion and 29 AA continuous deletion at position 481 and 533–561. Notably, GDHY strain had another 120 AA continuous deletion at position 629–748, which was similar with the MLV vaccine strain TJM derived from HP-PRRSV TJ strain and shared 99.8% identity with TJM (Fig. 2).

### Alignment and analysis of GP5 amino acid sequences

The GP5 AA sequences of all isolated strains were the same size with the reperesentative strains. AA sequence alignments showed that AA sequence identities among all the isolated strains ranged from 95.5–99.5%, with 86.1–89.1%, 91.0–92.5%, and 96.0–99.0% AA similarity with reference strains VR-2332, CH-1a and JXA1, respectively. They shared 85.2–95.4% AA identity with the NADC30 and recently isolated NADC30-like strains. However, they only had 56.9–58.4% AA identity with the European genotypic strain LV. These results indicated that all the isolated strains had more closer relationship with the North American genotype.

**Fig. 3:**
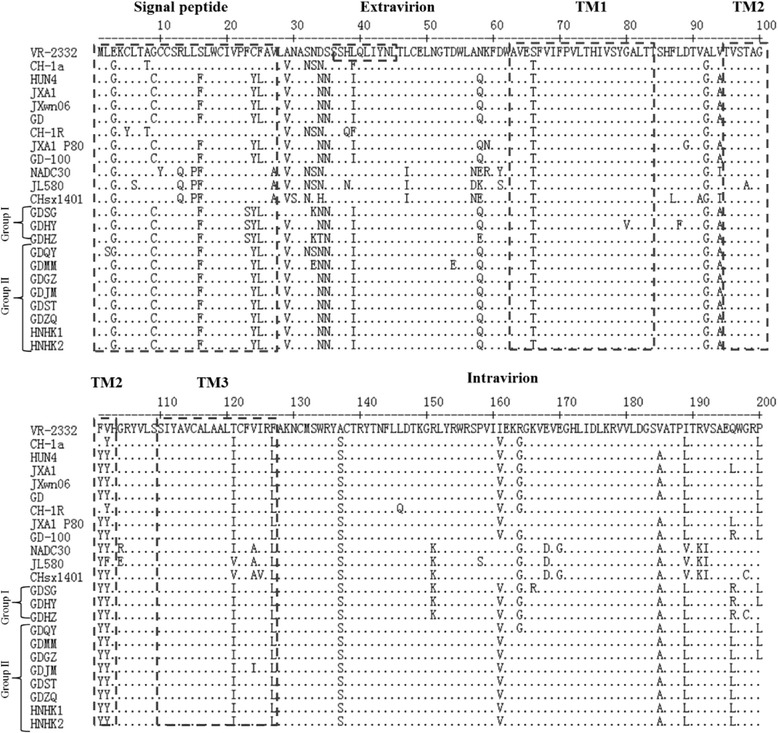
Alignment of the GP5 amino acid sequences of isolated PRRSV strains. The signal peptide, transmembrane region1, 2 and 3(TM1, TM2 and TM3), extravirion region, intravirion region and primary neutralizing epitope (PNE) were indicated by a *dotted box*

As Fig. 3 showed, the AA substitutions mainly focused on the putative signal, extravirion and intravirion region. The three transmembrane region (TM1, TM2 and TM3) were relatively conserved [16]. The residues R13 and R151 of GP5 are relevant to PRRSV virulence [17, 18]. All the isolated strains had the same AA R13 with the reference strain. However, in group I, GDHZ, GDHY and GDSG had a R151K residue mutation, which was identical to the NADC30 and NADC30-like strains (JL580 and CHsx1401). The residue A137 of GP5 was considered to differentiate vaccine strains (18). Compared with VR-2332, all the other reference and isolated strains in this study had an A137S mutation.

It was reported that the primary neutralizing epitope (PNE) of GP5 AA37–44 played a vital role in inducing immune responsiveness [19]. All the isolated strains had a L39I mutation, compared to the VR-2332 strain.

The N30, N34, N44 and N51 of four potential N-glycosylation sites of GP5 are related with viral infection and antigen characteristics [20]. In group I, only GDHZ had a N34 T mutation. All the other isolated strains had conserved N30, N44 and N51 glycosylation sites.

## Discussion

PRRSV has been one of the most prevalent diseases in pigs since its emergence in China, recently the NADC30 like strains have been extensively reported in most regions of China and it has caused huge economic loss to pig farmers (7–9). Considerable genetic diversity of PRRSV in field and invalid protection of current commercial vaccine to new emerging strains and in order to providing effect disease control, it is very necessary to execute frequent surveillance of the emerged new strains. In our study, 11 PRRSV strains were isolated from PRRSV positive samples in Guangdong and Hainan provinces of South China and the complete genomes were sequenced and analyzed. The results revealed that all the isolated strains were HP-PRRSV. Moreover, one isolated strain had another 120 AA continuous deletion except the discontinuous 30 (29 + 1) AA deletion in NSP2 region, which was similar with the MLV vaccine strain TJM derived from HP-PRRSV TJ strain [21].

Researchers have showed that recently isolated PRRSV strains belonged to North American strain and some strains had a close identity with NADC30 strain [22]. In our study, phylogenetic analysis indicated that all the isolated strains furtherly formed a subgenotype V with the representative strains. The subgenotype V was furtherly divided into two groups. GDHZ, GDHY and GDSG belonged to Group I, sharing a high homology with HP-PRRSV strains HuN4 and GD. The other isolated strains belonged to Group II, sharing a high identity with the HP-PRRSV strains JXA1 and JXwn06. No NADC30 like strains were isolated. These results indicated that the prevalent strain in Guangdong and Hainan was HP-PRRSV strains. Therefore, using the effective vaccine to resist the HP-PRRSV strain is a primary choose for controling HP-PRRS outbreak in these areas. Further surveillance should be reinforced to monitor the possibly emerging new strains.

NSP2 is the most variable region in the genome of PRRSV and was used for monitoring the evolution of PRRSV in the viral genome [23–25]. Comparing the NSP2 amino acid sequence of the 11 isolated strains with the reference strains VR-2332 and CH-1a, the NSP2 region of 10 isolated strains had 30 AA discontinuous deletion, including 1 AA deletion at position 481 and 29 AA continuous deletion at position 532 to 560. The result was identical with other Chinese isolated HP-PRRSV strains since 2006 [26]. Moreover, 1 strain GDHY had an another 121 AA continuous deletion at position 630 to 750 in NSP2 region, which was completely different from the previous reports (4–7, 17, 26). Compared with other isolated strains from pig farms, the clinic symptoms of pig infected GDHY was more severe. This result showed that a new extensive deleted PRRSV strain had emerged in Southern China. It is necessary to monitor the positive rate of PRRSV infected samples to determine whether this strain would be an epidemic strain in the future, so farmers can draw up a reasonable plan to control PRRSV outbreak.

GP5 is one of the most variant structural proteins in PRRSV, so it often been used to analyze viral genetic mutation [27, 28]. The PNE epitope and potential glycosylation sites of GP5 are related to the neutralizing activity, immune responsiveness, antigen characteristics and viral susceptibility (19, 20). Comparing to reference strain VR-2332, all the isolated strains had a L39I mutation in the PNE (AA37–44) epitope. In group I, only GDHZ had a N34 T mutation, which was different from the CH-1R and JXA1 P80 vaccine strains. All the other isolated strains had conserved N30, N44 and N51 glycosylation sites. It was reported that the AA changes of N-glycosylation sites in GP5 could benefit mutant virus escaping the neutralization [29]. At present, the attenuated modified live PRRS vaccine could not provide complete protection. Whether the failure of vaccine protection was relevant to the variation of N-glycosylation sites of GP5 will be further researched.

The residues R13 and R151 of GP5 are related to PRRSV virulence (15, 16). All the isolated strains had the same AA R13. However, in group I, three strains GDHZ, GDHY and GDSG had a R151K residue mutation, which was the same with the NADC30 and NADC30-like strains (JL580 and CHsx1401). It was reported that NADC30 like strain was virulent and extensively epidemic in China. Therefore, it might be that the change of R151 was relevant to the virulence of isolated PRRSV strains. Besides, our researches showed that AA mutation mainly focused on the putative signal, extravirion and intravirion regions of GP5. The changes of AA in these regions might affect the form of normal GP5 in cells and promote mutation of PRRSV [27, 28].

## Conclusion

In summary, we analyzed evolution characteristics of 11 PRRSV isolated strains in South China from 2014 to 2015. These results indicated that HP-PRRSV is still the prevalent strains in the region. One strain had another 120 AA continuous deletion except the discontinuous 30 (29 + 1) AA deletion in NSP2 region compared with the other isolated strains and reference strains. The GP5 of all isolated strains had many AA substitutions in the putative signal, extravirion, intravirion and extravirion regions. These results will benefit for vaccine research and disease control in this area.

## Abbreviations

AA: Amino acid
dNTP: Deoxynucleoside triphosphate
E: Envelope
HP-PRRSV: Highly pathogenic porcine reproductive and respiratory syndrome virus
M: Membrane
N: Nucleoprotein
NSPs: Nonstructural proteins
ORFs: Open reading frames
PRRS: Porcine reproductive and respiratory syndrome
PRRSV: Porcine reproductive and respiratory syndrome virus
S: Spike
